# A new bioluminescent cell-based assay for the detection of alpha-3 acetylcholine receptor antibodies: a multicentre comparison with current methods

**DOI:** 10.1186/s40478-026-02386-9

**Published:** 2026-08-08

**Authors:** Alex Stoyanov, John Tzartos, Hannah Fox, Maria Pechlivanidou, Peter Bradhurst, Valeria Iodice, Laura Watson, Giacomo Chiaro, Judith M. Spies, Stephen Adelstein, Patrick Waters, Nicolás Urriola

**Affiliations:** 1 Department of Clinical Immunology, Royal Prince Alfred Hospital, Level 6, Building 77, Vic Block, 50 Missenden Rd. Camperdown, Sydney, NSW Australia; 2 Central Sydney Immunopathology Laboratory, Royal Prince Alfred Hospital, Sydney, Australia; 3 Institute of Academic Medicine, Sydney Local Health District, Sydney, Australia; 4 Central Clinical School, Faculty of Medicine and Health, University of Sydney, Sydney, Australia; 5 Second Department of Neurology, Attikon University Hospital, National and Kapodistrian University of Athens, Athens, Greece; 6 Tzartos NeuroDiagnostics, Athens, Greece; 7 Nuffield Department of Clinical Neurosciences, University of Oxford, Oxford, UK; 8 University College London Hospital NHS Trust, London, UK; 9 Neurology Department, Royal Prince Alfred Hospital, Sydney, Australia

**Keywords:** Acetylcholine receptor antibodies, α3 antibodies, Ganglionic antibodies, Autoimmune autonomic ganglionopathy, Cell-based assays

## Abstract

Autoimmune autonomic ganglionopathy (AAG) is a disorder of the autonomic nervous system associated with autoantibodies against the α3 subunit of the ganglionic acetylcholine receptor (gnACHR). gnACHR antibodies exert their effects via receptor internalisation, but most current gnACHR diagnostic assays only detect antibody binding. Hence, we developed a new bioluminescent cell-based assay (BLIA) to measure internalising gnACHR antibodies. The BLIA was performed by incubating patient sera with live IMR-32 cells that express surface gnACHR. Remaining surface gnACHR was quantified using a novel split luciferase system with NanoBiT conjugated detecting antibodies. The percentage of receptor internalisation was calculated with comparison to cells incubated with reference sera, with > 20% receptor internalisation considered positive for gnACHR antibodies. The BLIA was validated using 46 samples from 20 AAG patients and 203 control samples. Subsequent comparisons to four existing assays were performed: (1) radioimmunoprecipitation assay (RIPA), (2) flow cytometric internalisation assay (FCIA), (3) cell-based assay (CBA-1) where HEK293T cells were transfected with α3β4 gnACHR subunits and (4) optimised CBA-2 with additional transfection of RIC3 and NACHO and incubation with 1 mM nicotine. The BLIA detected gnACHR antibodies with 100% sensitivity and 100% specificity (AUROC = 1.0) with detection of the internalising effects of antibodies to concentrations of  < 0.1 pM. There was complete qualitative agreement with CBA-2 (Cohen’s kappa = 1). No statistical difference between these two assays and the RIPA, which had slightly lower sensitivity (98%) and specificity (95%), or the FCIA, which had slightly reduced specificity (95%), was found. CBA-1 had significantly lower sensitivity (69%, *p* = 0.0003). Overall, the BLIA had perfect clinical sensitivity and specificity in detecting gnACHR antibodies in AAG patients and detection of the functional effects of gnACHR antibodies via internalisation offers a suitable alternative to current assays. Further assessment of its possible role in monitoring disease severity or treatment response is warranted.

**Supplementary Information:**

The online version contains supplementary material available at https://doi.org/10.1186/s40478-026-02386-9.

## Introduction

Autoimmune autonomic ganglionopathy (AAG) is a rare immune-mediated disorder of the autonomic nervous system that results in autonomic failure without motor or sensory deficits [1, 2]. Pathogenic autoantibodies against the α3 subunit of the ganglionic acetylcholine receptor (gnACHR) are found in a significant proportion of patients with AAG and likely act at the autonomic ganglion, resulting in downstream autonomic failure [3–5].

There are currently no consensus guidelines for the clinical diagnosis of AAG [6], but the accurate laboratory detection of gnACHR antibodies is key in distinguishing it from other forms of autonomic failure [7]. Timely and accurate detection of gnACHR antibodies is particularly important, as unlike other causes of autonomic failure, immunosuppressive treatments have been reported to be effective in AAG, and early diagnosis can prevent significant morbidity [8, 9].

Traditional assays to detect gnACHR antibodies, including the radioimmunoprecipitation (RIPA) and luciferase immunoprecipitation (LIPS) assays, detect autoantibody receptor binding [10]. Antibody levels determined by the former have been found to correlate with disease severity [11, 12]. However, both assays are limited by their lower specificity, with antibodies detected in a significant proportion of patients with non-immune mediated autonomic impairment [13], other neurological diseases [14, 15], autoimmune disease [16] and even healthy controls [17]. This poses significant clinical challenges, particularly in real world testing for this extremely rare and therefore low pre-test probability disease. Additionally, up to 50% of AAG patients have been reported to be “seronegative” due to a lack of detectable gnACHR antibodies by RIPA [18]. It is unclear if these patients have clinically relevant pathogenic antibodies of low affinity or concentration [6], or represent a distinct clinical entity [19, 20].

Attempts to develop live, transfected cell-based assays (CBAs) to detect gnACHR have previously been hindered by low surface expression of the receptor [21]. More recently, the first transfected CBA to detect gnACHR binding antibodies with high sensitivity and specificity has been described [22]. This assay requires transfection of the HEK293T cells with multiple plasmids encoding the α3 and β4 subunits of the gnACHR, the chaperone proteins NACHO (Nicotinic acetylcholine receptor regulator chaperone) and RIC3 (Resistance to inhibitors of cholinesterase 3), and addition of 1 mM nicotine to the culture medium to improve surface receptor expression. The signal is then amplified by secondary and tertiary antibodies [22].

gnACHR autoantibodies have been shown via electrophysiological and confocal imaging studies to exert their pathogenic effects via immunomodulation, with receptor cross-linking and internalisation leading to its removal from the cell surface upon autoantibody binding [23, 24]. The detection of the internalising effects of autoantibodies on surface gnACHR, rather than the detection of binding only, may therefore improve diagnostic accuracy. A flow cytometric assay using IMR-32 cells, a live-cell line with native surface receptor expression, has been established that detects internalising gnACHR autoantibodies with very high sensitivity and specificity [25]. The assay’s complex workflow and the long data acquisition times required for flow cytometric analysis, however, hinder its use as a high-throughput diagnostic test.

In this study, we developed a novel live cell-based bioluminescent assay (BLIA) that detects the internalisation of surface gnACHR on IMR-32 cells by serum autoantibodies, to support the diagnosis of AAG. The diagnostic performance of the assay was directly compared to the established flow cytometric internalisation assay, RIPA and two live CBAs, including the previously described optimised and high sensitivity CBA.

## Materials and methods

### Study cohort

**Fig. 1:**
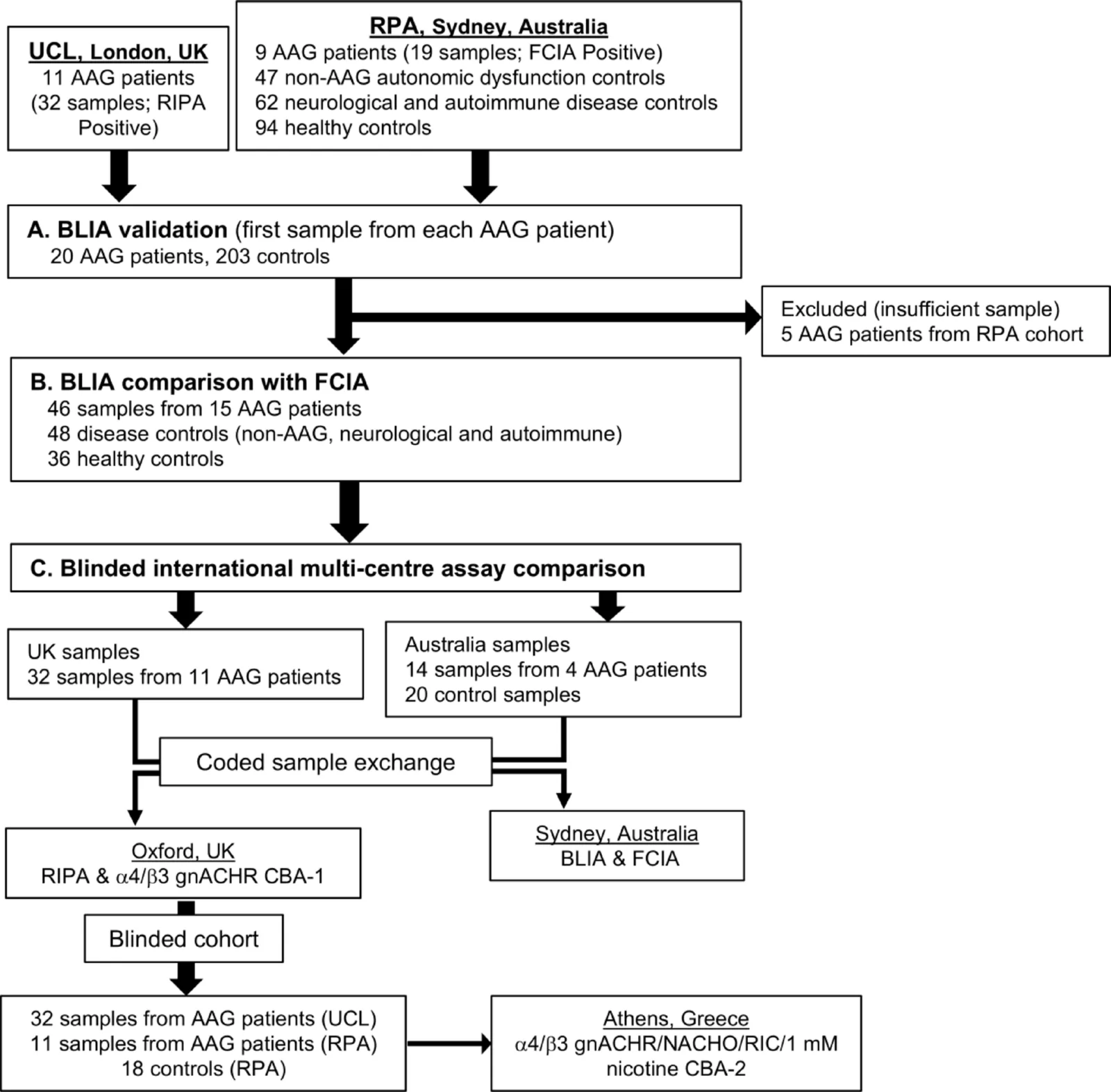
Validation and multicentre comparison of the BLIA with the RIPA, FCIA, CBA-1 and CBA-2. All AAG cases met published proposed diagnostic criteria [6] including pandysautonomia with orthostatic hypotension and detectable gnACHR antibodies via a locally established method (RIPA for the UCL cohort and flow cytometric internalisation assay for the RPA cohort). All control patients were collected at RPA Hospital (Sydney, Australia). Non-AAG cases had alternative causes for autonomic dysfunction identified (Table 1) and underwent comprehensive assessment in a quaternary clinic including autonomic function testing

The AAG cohort used to assess assay performance consisted of sera or defibrinated plasma from 9 patients with AAG (19 samples total) from the Royal Prince Alfred (RPA) Hospital’s Immunopathology Laboratory in Sydney, Australia and 11 patients with AAG (32 samples total) from the University College London (UCL) NHS clinic in London, UK (Fig. 1). Patients with AAG were defined according to the proposed diagnostic criteria for AAG [6]. All patients had autonomic failure that included impaired pupillary responses, orthostatic hypotension, impaired sweating and bowel and bladder dysfunction, corroborated by formal autonomic function testing, with other causes of autonomic failure excluded. All patients were gnACHR antibody positive via locally established assays; either by RIPA (> 100 pM) for the UCL cohort or for receptor internalisation via the flow cytometric internalisation assay (FCIA) for the RPA cohort.

Sera from 203 control participants including those with other causes of autonomic dysfunction (non-AAG; 47 samples), with neurological and systemic autoimmune diseases (disease controls; 62 samples) and healthy controls (94 samples), were obtained from RPA Hospital in Sydney. The characteristics of each control group are described in Table 1. All patients included in the non-AAG autonomic dysfunction group underwent comprehensive assessment with an autonomic specialist allowing accurate clinical characterisation and classification as having an alternative cause for autonomic dysfunction.

**Table 1:** Control group characteristics for BLIA assay validation

Control group	N (%)
***Autonomic dysfunction controls***	***47***
Autonomic failure secondary to systemic illness (e.g. diabetes mellitus)	14 (30%)
Postural orthostatic tachycardia syndrome (POTS)	10 (21%)
Isolated gastrointestinal dysmotility	7 (15%)
Pure autonomic failure	6 (13%)
Isolated postural hypotension	5 (11%)
Multiple system atrophy (MSA)	1 (2%)
Paraneoplastic pandysautonomia	1 (2%)
Ross syndrome	1 (2%)
Other causes of autonomic dysfunction	2 (4%)
***Disease controls***	***62***
Multiple sclerosis	9 (15%)
Paraneoplastic neurological syndromes	7 (11%)
Autoimmune encephalitis	6 (10%)
Myasthenia gravis	6 (10%)
Degenerative neurological disease (e.g. Parkinson disease)	5 (8%)
Neuromyelitis optica and optic neuritis	5 (8%)
Systemic vasculitis	5 (8%)
Systemic lupus erythematosus	4 (6%)
Peripheral neuropathy	3 (5%)
Rheumatoid arthritis	2 (3%)
Cryoglobulinaemia	1 (2%)
Other neurological disorders	5 (8%)
Other systemic inflammatory disorders	4 (6%)
***Healthy controls***	***94***

Each control group is denoted in bold

### BLIA validation

The earliest collected serum or defibrinated plasma sample from the 20 individuals with AAG and the 203 control samples were used to establish the performance characteristics of the BLIA (Fig. 1). Results from the healthy control group were used to establish a reference range and the decision threshold for determining positive gnACHR internalisation. Inter-assay variability was measured across more than 15 individual runs for the following: a strong gnACHR antibody positive AAG sample, a weaker gnACHR antibody positive AAG sample and a healthy control. The effect of interference on the BLIA assay was evaluated by measurement of the weak gnACHR antibody positive sample and healthy control sample spiked with grossly lipaemic, icteric and haemolysed sera respectively. The effect of intravenous immunoglobulin was assessed by spiking the same samples with pooled human immunoglobulin (10% Octagam and Privigen AU in a 1:10 dilution).

To determine the analytical sensitivity of the BLIA, all 20 AAG patient samples were serially diluted to determine the lowest serum dilution at which gnACHR internalisation remained detectable above the decision threshold.

### Comparison of BLIA with the flow cytometric internalisation assay (FCIA)

The BLIA and FCIA were compared blinded at the RPA Immunopathology Laboratory using 46 gnACHR antibody positive AAG samples (14 sera from 4 patients from the RPA cohort and 32 sera from 11 patients from the UCL cohort), 48 disease control and 36 healthy control samples. This cohort included additional samples taken at different disease and treatment timepoints from 15 of the 20 individual AAG patients used for the initial BLIA validation (Fig. 1). Five of the AAG samples from the RPA cohort were not tested due to insufficient sample.

### International multicentre gnACHR antibody assay comparison

The 46 AAG samples from the above comparison and 20 control samples (12 disease and 8 healthy controls) were then assessed by all five assays including the newly established BLIA, FCIA, RIPA, and two CBAs (1 and 2) for multi-assay comparison. Both CBAs utilised HEK293 cells transfected with α3 and β4 gnACHR subunits while the optimised CBA-2 also included transfection of RIC3 and NACHO chaperone proteins and treatment of cells with 1 mM nicotine to increase surface receptor expression; their respective methodologies are outlined in greater detail below [22]. BLIA and FCIA testing was performed at the RPA Immunopathology Laboratory (Sydney, Australia) while RIPA and CBA-1 testing was performed at the Nuffield Department of Clinical Neurosciences (Oxford, UK). CBA-2 was performed by Tzartos NeuroDiagnostics in Athens, Greece with samples from both cohorts blinded prior to analysis (Fig. 1). The 32 samples from 11 AAG UK patients in the UCL cohort (all RIPA positive) were sent coded to Australia for BLIA and FCIA testing, while 34 RPA samples (14 gnACHR antibody positive samples from 4 AAG patients and 20 controls) were sent to the UK for RIPA and CBA-1 testing. A post-hoc coded cohort from these two populations (32 AAG UK samples, 11 AAG Australia samples and 18 Australia control samples) were then sent from the UK to Greece for testing on the CBA-2.

### Statistical analysis

Results were used to calculate the sensitivity and specificity of the BLIA in the detection of gnACHR antibodies in AAG and receiver operating characteristics (ROC) analysis was conducted to determine the diagnostic accuracy of the assay.

Sensitivity and specificity of the tests were determined in the multi-assay comparison. McNemar’s test on paired proportions was performed to compare assay performance. Correlation and agreement of the BLIA with the four established assays were calculated using linear regression models (for quantitative values), Cohen’s kappa statistic calculations (for categorical values) and Bland–Altman analysis. All statistics were performed with Graphpad Prism 10 software.

### Assay methodologies

#### Bioluminescent cell-based assay for internalising gnACHR antibodies (BLIA)

IMR-32, a neuroblastoma live cell line that expresses functional surface gnACHR as demonstrated by prior immunofluorescence and electrophysiological experiments [26], was used for the novel bioluminescent assay. IMR-32 cells express fully formed surface gnACHR and hence no cell transfection or transduction was required. Cells were maintained in Eagle’s Minimum Essential Media (EMEM), supplemented with 10% foetal bovine serum (FBS), 2 nM glutathione, 1% non-essential amino acids and 100 IU/mL penicillin/100 µg/mL streptomycin at 37 °C with 5% CO2 and 95% humidity. Media was replenished every 48 h and cells were harvested for the assay at approximately 80% confluence in the logarithmic growth phase.

**Fig. 2:**
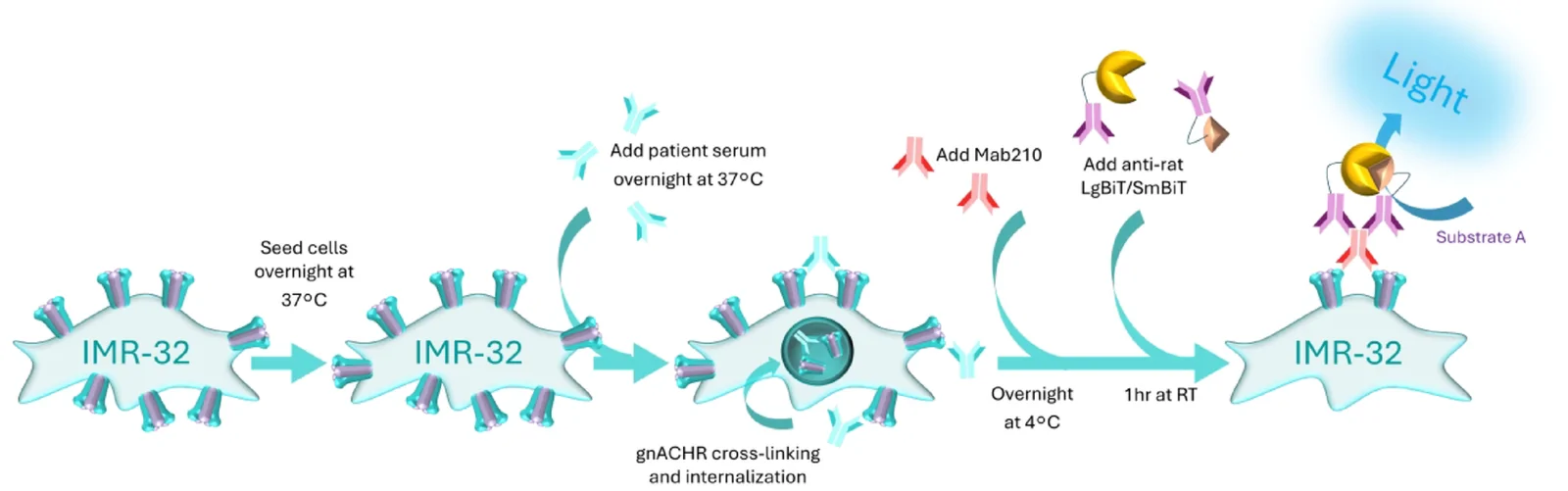
Novel bioluminescent internalisation assay (BLIA) for the detection of gnACHR autoantibodies. Patient sera were incubated overnight with IMR-32 cells which natively expresses functional gnACHR on the cell surface. gnACHR specific autoantibodies cross-link and internalise gnACHR overnight if present in patient serum. Residual surface receptor was then quantified using a split-luciferase NanoBiT detection system with a reduction in light signal (with comparison to pooled healthy reference sera) of > 20% occurring in the presence of internalising gnACHR autoantibodies

To perform the BLIA, cells were harvested using 0.25% trypsin (Sigma) and re-suspended in Roswell Park Memorial Institute (RMPI) media with 10% FBS before cell counting using a haemocytometer (Hirschmann, Ref 8,100,204). 3 × 10^5^ IMR-32 cells were then seeded in 190µL RPMI 1640 + 10% FBS media into a clear-bottom tissue culture 96-well plate. This seeding density allowed the cells to form a complete monolayer following the incubation steps outlined below. Cells were incubated overnight at 37 °C with 5% CO2 to allow cell re-attachment. Ten microlitres of sera (diluted 1:20 in media) were then added to individual wells and further incubated overnight at 37 °C (Fig. 2).

Subsequently, the supernatant was decanted and the cell-monolayer was washed once with wash buffer (Hanks Balanced Salt Solution, HBSS, with 1% FBS). 1% FBS was used in the wash buffer to minimise non-specific binding and enhance the bioluminescent signal at final reading. Remaining surface gnACHR was detected and quantified with the addition of 0.2 μg/mL primary antibody (Mab210 rat anti-gnACHR; Santa-Cruz Biotechnology cat#: sc-58603) [27] in 100μL wash buffer, incubated for 24 h at 4 °C. Background or blank wells to determine background luminescent signal and matrix effects from FBS were incubated with reference sera but had no primary antibody added. Cells were then washed twice with wash buffer and incubated with NanoBiT labelled anti-rat secondary antibody (SouthernBiotech cat#:3051 conjugated in-house according to the manufacturer instructions using the Lumit® labelling kit; Promega Corporations, cat#: N2014) in 100μL wash buffer for 60 min at room temperature (RT). The NanoBiT system utilises a split luciferase in which the NanoLuc enzyme is split into two components, a large (LgBiT) and small (SmBiT) component, with reconstitution of the whole NanoLuc and enzymatic activity only occurring when the two components are in proximity. In the BLIA, secondary anti-rat antibodies were conjugated to SmBiT and LgBiT and added in a 1:1 ratio to give a total of 0.5 μg/mL anti-rat secondary antibody (Fig. 2). Furimazine-based substrate (Substrate A, Promega Corporations cat#:VB2020) was then added at a final concentration of 1:1000 and the luminescent signal (in relative light units, RLU) was read at 75 min using the PerkinElmer Enspire multiplate reader, with an acquisition time of 0.1 s per well.

The degree of gnACHR receptor internalisation was determined by comparing the light signal from the patient sample with the light signal from wells incubated with a pool of reference healthy control sera after accounting for background signal using the formula below. Serum that produced a reduction in light signal of 20% or greater was considered positive for gnACHR antibodies with the rationale for selection of this cut-off outlined in the Results section below.

$$\begin{aligned} gnACHR\;internalisation\left( \% \right) = & 100 \times \left[ {1 - \left( {\frac{{Sample\;RLU - Background\;RLU}}{{Reference\;sera\;RLU - Background\;RLU}}} \right)} \right] \\ \end{aligned} $$

### Flow cytometric internalisation assay (FCIA)

Detection of gnACHR antibodies by flow cytometry was performed via a previously published method with minor modifications and is outlined in greater detail in the Supplementary Material including Supplementary Fig. 1 [25]. Briefly, patient sera were incubated overnight with IMR-32 cells in RPMI 1640 + 10% FBS. Cells were washed twice, fixed with 1% paraformaldehyde, washed again to remove excess fixative and then stained with rat anti-gnACHR antibody (Mab35, Sigma cat#: 217). Cells were washed again before staining with anti-rat IgG conjugated to phycoerythrin (Southern Biotech cat#: OB3030-09) and resuspension in 1% paraformaldehyde prior to flow cytometric analysis. A reduction in detectable surface gnACHR of greater than 18% via flow cytometric analysis was defined as positive for gnACHR internalising antibodies.

### Radioimmunoprecipitation assay (RIPA)

RIPA was performed using the RiaRSR™ gAChR Ab kit as per manufacturer instructions. Briefly, 50μL solubilised recombinant ^125^I labelled gnACHR was incubated with 10μL patient sera (1:6 dilution) for 2 h at RT. Immune complexes were precipitated via the addition of 50μL anti-human IgG for 2 h at RT. Immunocomplexes were then precipitated via the addition of 1 mL cold wash buffer followed by centrifugation at 15000xg for 5 min. Pellets were washed once in cold wash buffer and residual radioactivity counted using a gamma counter to determine gnACHR concentrations. Samples quantified at less than 10 pM were considered negative, samples of 10–15 pM were considered equivocal and samples greater than 15 pM were considered positive for gnACHR antibodies.

### Transfected live cell-based assay 1 (CBA-1)

HEK293T cells cultured on poly-l-lysine coated glass coverslips were transiently transfected with human α3 and β4 gnACHR subunits. After 24 h, the cells were incubated with patient sera at 1:20 dilution for 1 h at room temperature. Coverslips were washed thrice in DMEM/HEPES and fixed for 5 min in 4% formaldehyde, after which cells were incubated with an unconjugated goat anti-human IgG (Fc) antibody for 45 min (cat#: 31,125, Invitrogen). Coverslips were again washed thrice and incubated with Alexa Fluor 568 conjugated donkey anti-goat IgG (H + L) for 45 min (cat#: A11011, Invitrogen). Coverslips were washed four times and mounted onto DAPI supplemented fluorescent mounting media on glass slides. Coverslips were read by two independent readers using a fluorescent microscope (DM2000, Leica Microsystems) to determine the presence or absence of bound surface gnACHR antibodies. Negative samples had a fluorescence reader score of less than 1 while positive samples scored 1 to 4 based on staining intensity. The methodology for CBA-1 is outlined in greater detail in the Supplementary Materials including Supplementary Fig. 2.

### Optimised transfected live cell-based assay 2 (CBA-2)

As previously published [22], HEK293T cells were transiently co-transfected with a mixture of plasmids for the α3 and β4 gnACHR subunits, RIC3 and NACHO chaperone proteins and treated with 1 mM nicotine for 24 h to maximise surface receptor expression. Forty-eight hours post-transfection, cells were washed and then incubated with patient sera at 1:10 dilution for 1 h, followed by fixation and visualisation (microscopy analysis by two independent observers) after secondary and tertiary antibody incubation at RT. The methodology for CBA-2 is outlined in greater detail in the Supplementary Materials including Supplementary Fig. 3.

## Results

### Novel BLIA validation and diagnostic performance

**Fig. 3:**
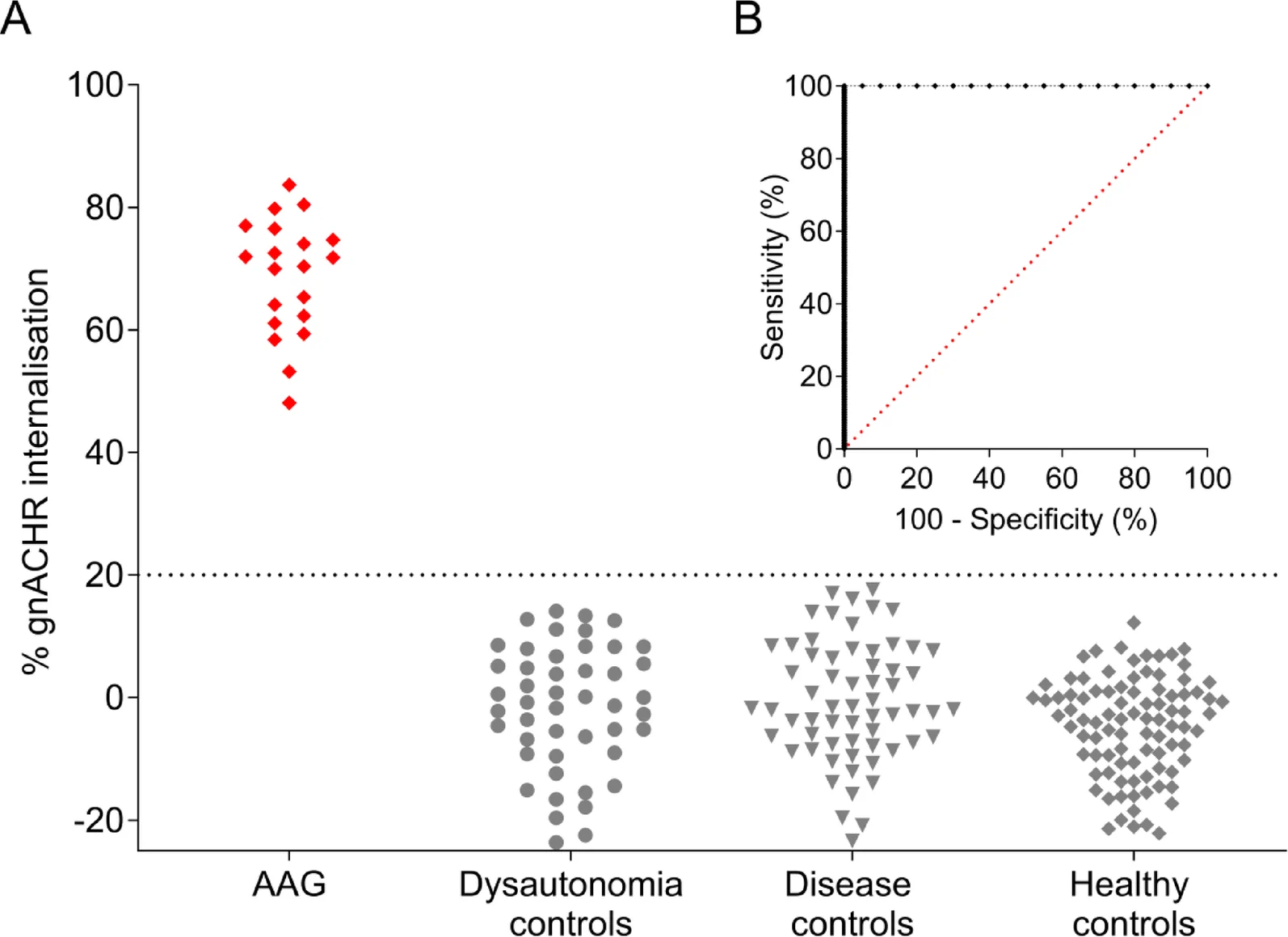
Diagnostic performance of novel bioluminescent internalisation assay (BLIA) for detection of gnACHR antibodies. **A** A cut-off of 20% gnACHR internalisation identified AAG patients with 100% sensitivity and 100% specificity. Groups: AAG (n = 20), dysautonomia controls (n = 47), disease controls (n = 62) and healthy controls (n = 94) and **B** Receiver Operator Characteristic (ROC) curve analysis demonstrated perfect discrimination between AAG and non-AAG samples using the BLIA (AUROC = 1)

A decision threshold of 20% gnACHR receptor internalisation for gnACHR antibody detection was calculated based on the mean gnACHR internalisation of 94 healthy control samples plus 3 standard deviations (Mean internalisation − 4.5%, standard deviation 8.0%). Based on this cut-off, all 20 AAG patient samples were considered BLIA positive (Mean internalisation 68.7%, standard deviation 9.4%, range 48–84%) and all 203 controls were negative, giving the BLIA 100% sensitivity and 100% specificity in the validation cohort. All AAG samples demonstrated receptor internalisation of greater than 45% (equal to the mean healthy control gnACHR internalisation + 6 standard deviations). No disease or healthy control sera resulted in greater than 20% internalisation (Fig. 3A). A ROC curve analysis showed perfect discrimination between AAG and non-AAG samples with an area under the ROC (AUROC) of 1.00 (Fig. 3B). ROC curve analysis also confirmed 100% sensitivity and specificity using the calculated cut-off of 20% receptor internalisation.

No significant receptor internalisation was observed in the presence of interfering substances including gross lipaemia, haemolysis or hyperbilirubinaemia. When sera with these interfering substances was spiked into AAG patient sera, gnACHR internalisation remained detectable above the decision threshold (Supplementary Fig. 4). Intravenous immunoglobulin did not alter the gnACHR internalisation seen in an AAG sample nor cause a false positive result in healthy control serum. Comparison of more than 15 separate assay runs for three samples (2 AAG patients and a healthy control) demonstrated an inter-assay standard deviation of between 5 and 6% receptor internalisation corresponding to a %CV (co-efficient of variation) of 8% and 14% for the two AAG samples respectively (Supplementary Fig. 4). This assessment included different batches of IMR-32 cells, conjugated secondary detecting antibodies and detecting substrate.

The BLIA demonstrated high analytical sensitivity with gnACHR internalisation detectable above the decision threshold at dilutions of either 1:2560 or 1:5120 for all individual gnACHR positive AAG patients. This corresponded to antibody concentrations of 0.03–0.07 pM as measured by RIPA for the 15 samples in the comparison cohort for which current RIPA results were available (Supplementary Table 1).

### Comparison of the BLIA with the flow cytometric internalisation assay (FCIA)

**Fig. 4:**
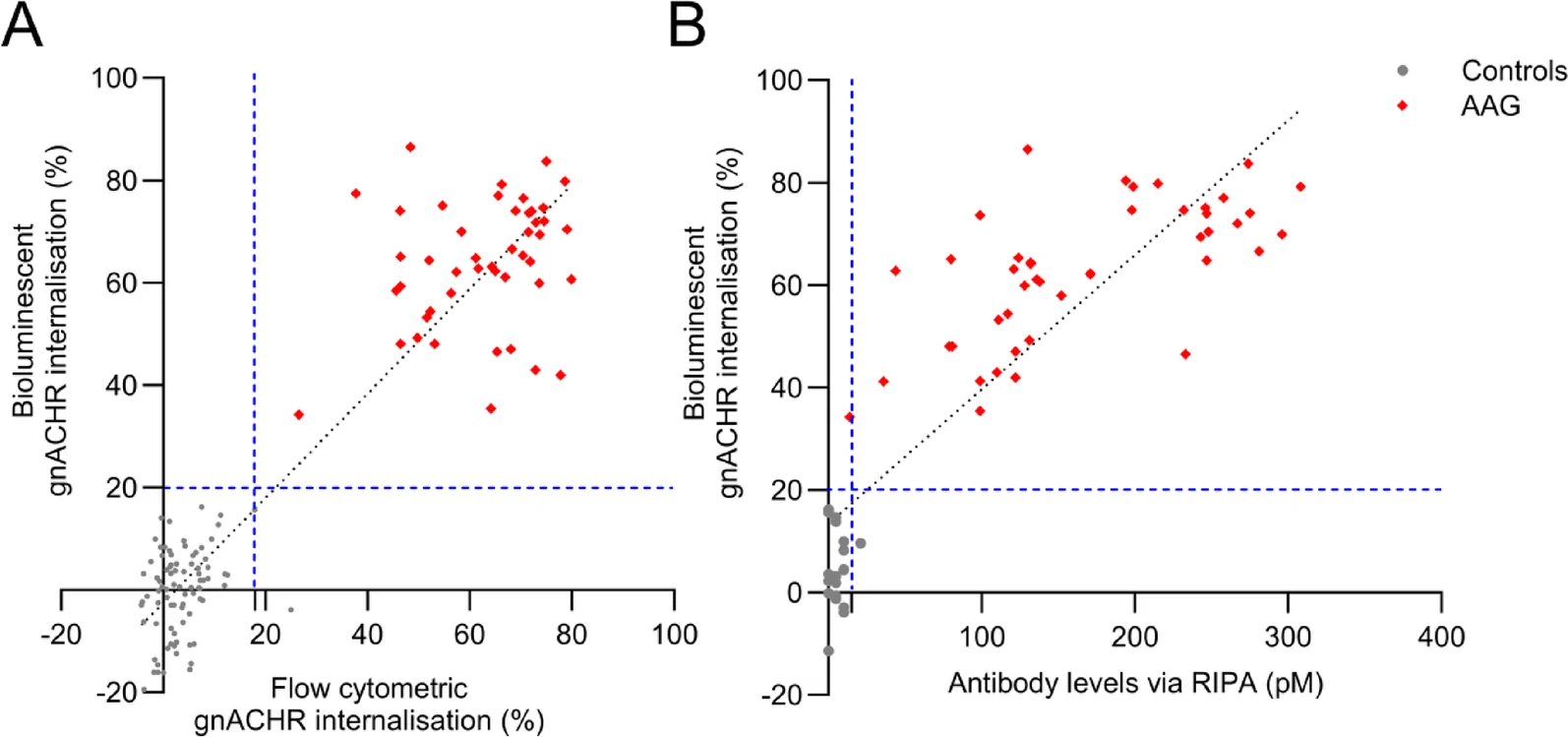
Correlation between the BLIA, FCIA (% internalisation) and RIPA (gnACHR antibody concentration). **A** A high level of correlation was observed between the BLIA and FCIA for 130 analysed samples (46 AAG patients and 84 controls) (R^2^ = 0.88). Both assays had 100% sensitivity; the BLIA had 100% specificity while the specificity for the FCIA was 98.8% with a positive result detected in a single control. Cut-offs for both assays are denoted by the blue dashed lines. **B** gnACHR internalisation correlated with gnACHR antibody concentrations via RIPA (R^2^ = 0.75) for 66 samples (46 AAG and 20 control samples)

Forty-six samples from the 15 AAG patients used for assay comparison had detectable gnACHR receptor internalisation via both the BLIA and FCIA with both assays demonstrating 100% sensitivity. The BLIA had 100% specificity compared to 98.8% for the FCIA (Fig. 4A and Supplementary Table 2). Low level gnACHR internalisation was detected via the flow cytometry assay in one autonomic dysfunction control with isolated orthostatic hypotension without additional clinical features of autonomic failure. This sample had no gnACHR antibodies detected via RIPA or CBA-2 in the multi-assay comparison below.

There was a high level of qualitative agreement (Cohen’s kappa statistic, K = 0.98) between both assays in distinguishing AAG from control samples (Table 2). Quantitative receptor internalisation results from both assays demonstrated high levels of correlation (R^2^ = 0.88) (Fig. 4A) and similarly, Bland–Altman analysis also revealed a high level of quantitative agreement between the two assays with a mean difference of only 1.9% receptor internalisation (95% CI: − 20.3 to 24.3% receptor internalisation) (Supplementary Fig. 5).

**Table 2:** Diagnostic performance, agreement and correlation between the BLIA and established comparator assays

Assay	Sensitivity (n = 46)	Specificity (n = 20)	Agreement (K)	Correlation (R^2^)	*p*-value
Bioluminescent internalisation assay (BLIA)	100% (46/46)	100% (20/20)	N/A	N/A	N/A
Flow cytometric internalisation assay (FCIA)	100% (46/46)	95% (19/20)	0.98	0.88	0.96
Radioimmunoprecipitation assay (RIPA)	98% (45/46)	95% (19/20)	0.92	0.75	0.47
CBA-1 (α3β4 transfected HEK293T cells)	69% (32/46)	100% (20/20)	0.57	N/A	0.0003**
Optimised CBA-2 (α3β4, RIC3, NACHO transfected HEK293 cells with nicotine)	100% (43/43)	100% (18/18)	1.0	N/A	1.0

Agreement between the BLIA and the comparator assay was calculated using Cohen’s kappa statistic for qualitative results and correlation was determined using linear regression models for assays with quantitative results. McNemar’s test was used to compare sensitivity of the BLIA with the comparator assay with p-values expressed in the final column (**indicates significantly lower sensitivity). CBA-2 results were unavailable for 3 AAG samples and 2 controls samples

### Multi-assay comparison

**Fig. 5:**
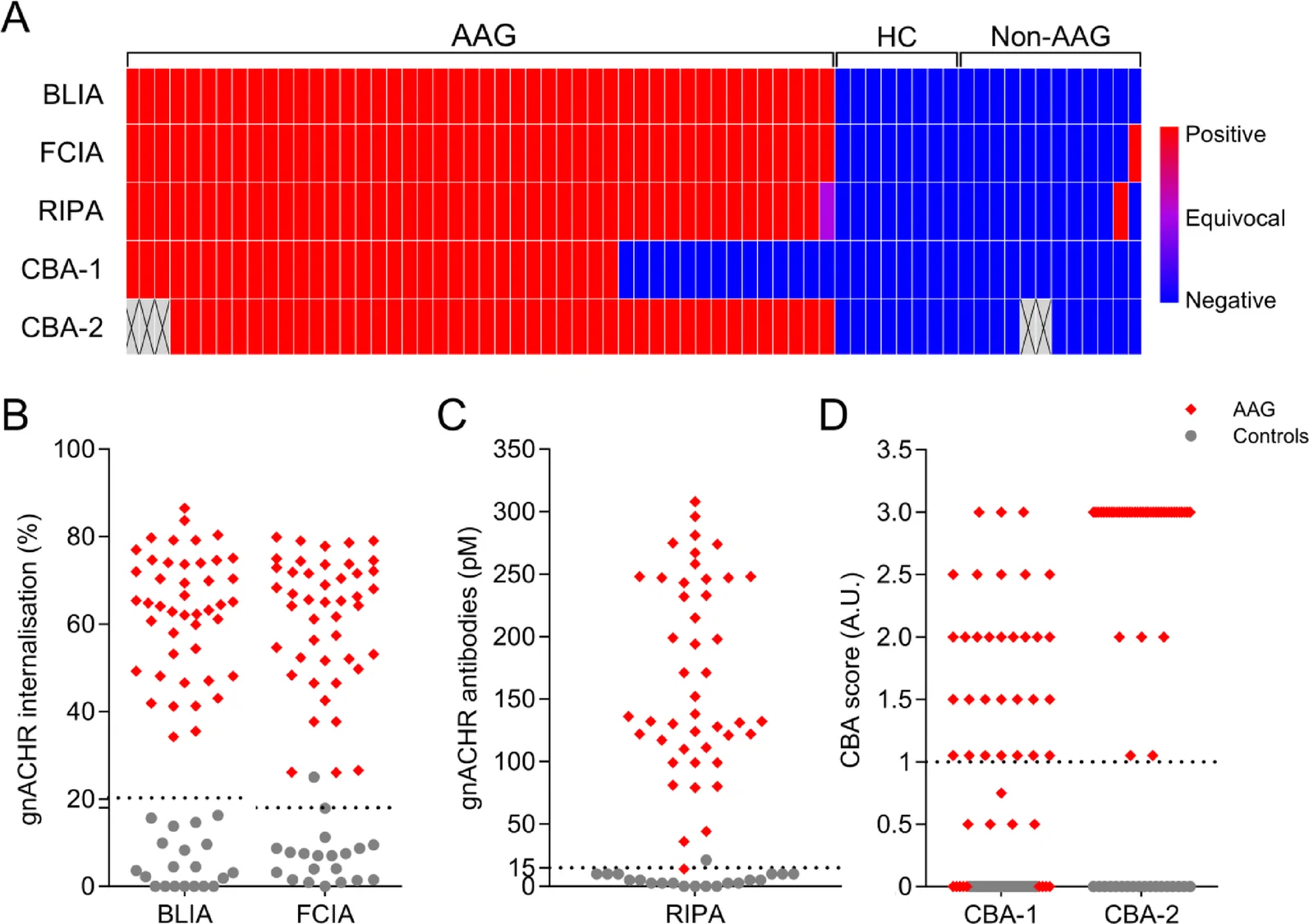
Comparison of gnACHR antibody detection via the BLIA and comparator assays. **A** Qualitative comparison for 46 AAG samples and 20 control samples with positive results denoted in red, equivocal in purple and negative in blue. Results for CBA-2 were not available for three AAG and two control samples. **B** Quantitative comparison of the BLIA with the FCIA, **C** RIPA and **D** CBA-1 and CBA-2. Measurement units for each assay are shown on the respective y-axes and assay cut-offs via the dotted lines. Results for CBA-2 were not available for three AAG and two control samples

The novel BLIA had high levels of qualitative agreement with all assays (Table 2 and Fig. 5A), (K > 0.9 for all assays) except for CBA-1. There was perfect agreement (K = 1.0) between the bioluminescent assay and the optimised transfected CBA (CBA-2) with both assays having 100% sensitivity and 100% specificity for the detection of gnACHR antibodies in the comparator cohort (Fig. 5).

CBA-1 had poorer sensitivity (69%), which was significantly lower than the BLIA (*p* = 0.0003) despite remaining highly specific. It failed to detect gnACHR binding in 14/46 (30%) AAG samples including several samples with high RIPA values (> 200 pM) and gnACHR internalisation of > 60% on both internalisation assays.

There was a high level of qualitative agreement between the BLIA and RIPA (K = 0.92). Similarly, quantitative gnACHR internalisation via the BLIA correlated with gnACHR antibody concentrations via the RIPA (R^2^ = 0.75) (Fig. 4B). The sensitivity of the RIPA was not statistically significantly lower than the BLIA or CBA-2, but it failed to detect gnACHR antibodies above the decision threshold in a sample from a treated AAG patient with ongoing symptoms. This sample had low level detectable gnACHR internalisation (34% receptor internalisation) using the BLIA and was also positive via the FCIA and CBA-2 assays. Similarly, the RIPA also returned a false positive result in a patient with an unspecified systemic inflammatory condition with marked polyclonal hypergammaglobulinaemia and no features of autonomic dysfunction. This sample was negative for gnACHR antibodies in all the other assays.

## Discussion

We present a novel bioluminescent cell-based gnACHR internalisation assay (BLIA) which detected gnACHR antibodies in 46 longitudinal samples from 20 patients with AAG with 100% sensitivity and 100% specificity. The diagnostic performance of the BLIA was compared to the established FCIA, RIPA and two transfected CBAs (CBA-1 and CBA-2) in the largest cross-comparison of diagnostic AAG assays published to date. All assays displayed high sensitivity and specificity, except for CBA-1, which had lower sensitivity than the other assays. There was a high level of diagnostic agreement between the BLIA and the comparator assays, including perfect agreement with CBA-2.

The RIPA detects binding of antibodies to solubilised gnACHR derived from IMR-32 and has a simple workflow that provides a quantitative readout which is useful for routine diagnostic laboratories. However, its specificity is poor when cut-offs of 20–50 pM are employed, with false positive results frequently observed in various neurological and non-neurological disorders [28, 29]. Higher decision limits (> 200 pM) increase specificity for AAG [30], although false positives still occur, with additional studies suggesting that the RIPA does not effectively predict the presence or absence of autonomic failure in the real-world setting [31, 32]. Whilst our study was not sufficiently powered to statistically assess specificity, we found that the RIPA detected gnACHR antibodies in a patient with systemic inflammation and polyclonal hypergammaglobulinaemia with no symptoms of autonomic dysfunction whilst the BLIA and CBAs were 100% specific. Our validation of the BLIA, and prior studies demonstrating the high specificity of the FCIA and CBA-2, have utilised large well-characterised control patient cohorts [22, 25], but direct comparison of specificity between assays has not previously been performed and was limited in our study. This should be the focus of future studies, including more detailed assessment of “RIPA false positive” samples via the cell-based assays presented here.

In contrast to live IMR-32 cells which express native surface gnACHR, prior attempts at the use of transfected cell lines for gnACHR detection have been limited by low surface receptor expression and uncertain subunit stoichiometry in transfected cell-based systems causing impairment of autoantibody binding and leading to reduced assay sensitivity [21, 33]. This was seen in CBA-1 in our study which utilises transfection of α3 and β4 subunits only and had poor sensitivity, detecting gnACHR antibodies in less than 70% of AAG patient samples. The addition of the chaperone proteins RIC3 and NACHO [34, 35] and a nicotine incubation step (CBA-2 in this study) to increase surface gnACHR expression has been described [22]. It has been shown to detect gnACHR antibodies effectively and like the BLIA, displayed 100% sensitivity and specificity in our study, with complete qualitative agreement between the two assays. Our study confirms previous data that the optimisation of transfection conditions in CBA-2 effectively increases expressed surface gnACHR to detect binding antibodies with at least equivalent sensitivity to the RIPA. This CBA offers a semi-quantitative result via end-point titres, has high specificity, and could be established in diagnostic laboratories experienced with live cell-based assays. However, it is potentially labour intensive, requires access to cell-culture facilities, and requires transfection at the commencement of every assay run [22].

The pathogenicity of gnACHR autoantibodies via internalisation, with receptor cross-linking and removal from the cell surface for lysosomal degradation, has been previously demonstrated in patch-clamp analysis and confocal microscopy studies [23, 24]. Detection of gnACHR antibodies via internalisation may therefore detect the functional pathogenic effects of autoantibodies and offers an alternative to the antibody binding detected via the RIPA and CBAs. Both receptor internalisation assays in this study, including the novel BLIA, demonstrated perfect sensitivity and high specificity with high levels of agreement with the RIPA and optimised CBA-2. In contrast to CBAs, the IMR-32 cells used in the BLIA and FCIA express stable high-density surface gnACHR [26] with minimal maintenance requirements and without the need for regular transfections. Both internalisation assays also minimise the background binding of irrelevant antibodies to the cell surface (which may cause difficulties in observer interpretation in the CBAs) and offer a quantitative measure in contrast to the semi-quantitative results yielded by the CBAs.

Autoantibody levels have been demonstrated to correlate with disease severity with higher levels via the RIPA associated with more severe autonomic failure [12, 36] and a decrease reported in some cases of treatment response [37]. The BLIA showed strong quantitative correlation with the RIPA and analysis of serial dilutions of the AAG samples via the BLIA demonstrated an analytical sensitivity of detecting below 0.1 pM of gnACHR autoantibody (as determined by quantified antibody concentrations via RIPA for each sample). While this was not determined separately for the RIPA and the CBAs in this cohort; prior published detection limits are 8 pM for CBA-2 and 10 pM for the RIPA [22]. The RIPA also failed to detect gnACHR antibodies in a treated patient with ongoing symptoms that remained detectable via the BLIA in our study. In addition, the RIPA strictly measures the concentration of precipitated receptor rather than receptor bound antibody which does not account for polyclonal autoantibody responses. Using the RIPA autoantibody concentrations to calculate sensitivity is therefore likely to underestimate the true analytical sensitivity of the BLIA and CBA-2 but still allows for some comparison between the assays. Further studies to assess whether ability of the BLIA to detect very low concentrations of gnACHR antibodies has a potential role in monitoring disease activity, response to treatment and earlier detection of disease relapse should therefore be considered.

The relative advantages and disadvantages of the diagnostic assays compared in this study are further outlined in Table 3 and many of these have been discussed above. The novel BLIA has several potential additional advantages including: (1) the bioluminescence detection system allows for rapid signal acquisition with the ability for 100 samples to be read in 10 s in an automated manner, (2) it provides a quantitative signal with an objective measure of receptor internalisation, in contrast to flow cytometry where gating can significantly affect results, and the CBAs which provide a visual interpretation of cell surface intensity which at best can be made semi-quantitative by end-point titration and (3) the use of the split luciferase system detects specific receptor binding [38] and significantly reduces the assay complexity with only three simple wash steps required, in contrast to the multiple wash steps required for the FCIA and CBAs. In addition, the BLIA along with the FCIA and CBA, do not have the safety concerns associated with the use of radioligands in the RIPA.

**Table 3:** Advantages and disadvantages of diagnostic assays for the detection of gnACHR antibodies in AAG

Assay	Advantages	Disadvantages
Bioluminescent internalisation assay (BLIA)	High clinical sensitivity and specificity Excellent analytical sensitivity (< 0.1 pM) Rapid signal acquisition time Simple workflow with reduced assay complexity due to use of split luciferase system	Requires equal cell numbers and cell growth conditions per well Takes 72–96 h to perform May theoretically detect blocking antibodies
Flow cytometric internalisation assay (FCIA)	High sensitivity and specificity Provides quantitative measure of receptor internalisation	Long signal acquisition time Subject to gating variability Complex workflow with multiple wash steps Takes 72 h to perform
Radioimmunoprecipitation assay (RIPA)	Simple workflow Provides result within hours Provides quantitative measure of antibody/receptor concentration	Variable specificity in prior studies depending on decision limit used with false positive rate Requires use of radioligands
CBA-1 (α3β4 transfected HEK293T cells)	Utilises simple cell transfection	Requires cell culture and transfection expertise and instrumentation Poor clinical sensitivity significantly limiting clinical utility Provides only semi-quantitative result
Optimised CBA-2 (α3β4, RIC3, NACHO transfected HEK293T cells with nicotine)	High clinical sensitivity with optimised transfection conditions Excellent specificity Reproducible workflow for laboratories experienced in CBAs	Requires cell culture and transfection expertise and instrumentation Provides only semi-quantitative result Takes 72–96 h to perform

There are a few possible limitations to the new BLIA. The mAb210 primary antibody used may be blocked by gnACHR antibodies directed to the same epitope. While prior studies have demonstrated that blocking antibodies to the epibatidine binding site of gnACHR are associated with autonomic failure [11], the potential significance of blocking antibodies to this epitope are unclear. In addition, mAb210 also binds to the α5 subunit of gnACHR but its expression is low in IMR-32 and unlikely to significantly affect results [27].

This study had several limitations. Firstly, the sample size of gnACHR antibody positive AAG patients was small. AAG remains a very rare disease, as demonstrated in other studies assessing clinical and diagnostic parameters that have also utilised small sample numbers. Although we only assessed 20 distinct patients with AAG, these were well characterised with supporting clinical and autonomic testing data and analysis of additional samples from a subset of AAG patients allowed further assessment of the sensitivity of the BLIA and comparator assays. Secondly, comparator assay results were available for only a small portion of the control patient cohort which limited comparison of assay specificity as discussed above. Thirdly, comprehensive clinical data was not available for the subsequent serial sample collections from AAG patients to allow the assessment of the assays in monitoring treatment response and disease activity, and this should be considered in future studies.

## Conclusions

The novel bioluminescent gnACHR internalisation assay (BLIA) presented in our study detected gnACHR antibodies with 100% sensitivity and 100% specificity and demonstrated very high analytical sensitivity. It had comparable diagnostic performance and high levels of agreement to comparator assays in the largest cross-comparison of AAG diagnostic assays to date. This study demonstrates that assays to detect the functional effects of gnACHR antibodies via internalisation offer a suitable alternative to current assays that detect antibody binding. The newly developed BLIA and optimised CBA (CBA-2) both had perfect clinical sensitivity and specificity; both have excellent applicability in the diagnostic laboratory for the diagnosis of AAG and we would recommend that laboratories offering gnACHR antibody detection adopt similar methods in the future. Further studies examining the specificity of these assays in greater detail, as well as their potential role in monitoring disease activity and treatment are warranted.

## Supplementary Information

Below is the link to the electronic supplementary material.

Supplementary Material 1

Supplementary Material 2

Supplementary Material 3

Supplementary Material 4

Supplementary Material 5

Supplementary Material 6

## Data Availability

The datasets used and/or analysed during the current study are available from the corresponding author on reasonable request.
