# Supplementary material for: A new bioluminescent cell-based assay for the detection of alpha-3 acetylcholine receptor antibodies: a multicentre comparison with current methods
Source: Acta Neuropathol Commun. 2026 Aug 8;14:228. doi: 10.1186/s40478-026-02386-9 (PMC13737872; doi:10.1186/s40478-026-02386-9)
Supplement: Supplementary file 1 [file 40478_2026_2386_MOESM1_ESM.pdf]

Supplementary Material

1. Flow cytometric internalisation assay (FCIA)

Detection of gnACHR antibodies by flow cytometry was performed via a previously published method with minor modifications and is outlined in **Supplementary Figure 1** below [25]. This assay also utilises live IMR-32 cells and cell handling measures were the same as those used for the BLIA.

To perform the flow cytometric assay, IMR-32 cells were seeded at a density of $5x10^5$ cells in 190µL RPMI + 10% FBS into a clear-bottom tissue culture 96-well plate to allow formation of a cell monolayer and incubated overnight at 37°C for cell re-attachment. Ten microlitres of sera (diluted 1:20) were then added to individual wells and further incubated overnight at 37°C. Ten microlitres of FBS were added to two wells to serve as controls for autofluorescence and maximally stained cells.

Cells were subsequently decanted gently and washed twice in 200µL wash buffer (Hanks Balanced Salt Solution, HBSS, with Ca^{2+} and Mg^{2+}, 1% FBS) before fixation with 100µL 1% paraformaldehyde in wash buffer for 15 minutes at 4°C. Cells were washed twice again to remove excess fixative and then stained with 100uL rat anti-gnACHR antibody (Mab35, Sigma cat#: 217) at 2µg/mL at 37°C for 30 minutes. Cells were washed a further two times, then stained with 100µL of a 1:1000 dilution of a secondary antibody (anti-rat IgG, conjugated to phycoerythrin, Southern Biotech cat#: OB3030-09), and left to incubate at room temperature for 30 minutes. No secondary antibody was added to background or “minimum” wells to assess for autofluorescence. Cells were washed twice more, then resuspended in 1% paraformaldehyde to a final volume of 200µL until analysis.

Cells were transferred to FACS tubes and analyzed on a BD FACSCANTO II flow cytometer. Gating was set to exclude non-viable events (prior to fixation), with subgating set around the discrete cell population evident on a forward and side scatter plot. Approximately 10,000 events of interest were counted in each test. gnACHR internalisation was calculated based on gnACHR positive events in patient samples with comparison to unstained (MIN) and maximally stained (MAX) cells using the formula below:

$$gnACHR\ internalisation\ (\%) = 100 \times \left[1 - \left(\frac{Sample\ events - MIN}{MAX - MIN}\right)\right]$$

A reduction in detectable surface gnACHR of greater than 18% via flow cytometric analysis was defined as positive for gnACHR internalising antibodies based on a previously published validation of this assay [25]. This assay has previously been validated with the assessment of 8 AAG patients positive for gnACHR via RIPA and 182 control patients [25].

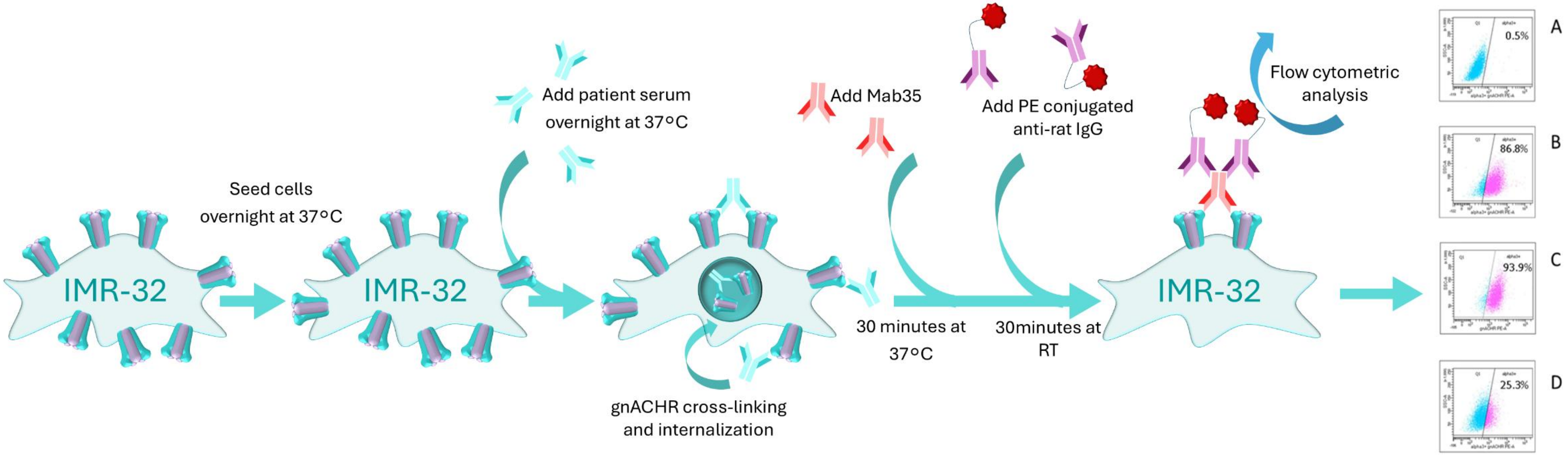

Supplementary Figure 1. Flow cytometric assay (FCIA) for the detection of internalising gnACHR autoantibodies. Patient sera were incubated overnight with IMR-32 cells with remaining surface gnACHR then quantified via flow cytometric analysis. Plots demonstrating (A) Background or minimum staining, (B) Maximally stained IMR-32 cells, (C) IMR-32 cells incubated with control sera with no reduction in surface gnACHR and (D) IMR-32 cells incubated with sera from an AAG patient with a significant reduction in surface gnACHR staining due to autoantibody mediated receptor internalisation.

2. Cell-based assay 1 (CBA-1)

CBA-1 was performed using HEK293T cells transfected with only the α3 and β4 subunits of the gnACHR and the methodology is outlined in **Supplementary Figure 2** below.

HEK293T cells were cultured on poly-l-lysine coated glass coverslips in Dulbecco's modified Eagle's medium (DMEM) and transiently transfected with human α3 and β4 gnACHR subunits using plasmids. After 24 hrs of incubation, the cells were further incubated with patient sera at 1:20 dilution for 1 hour at room temperature. Coverslips were then washed three times in DMEM/HEPES and fixed for 5 min in 4% formaldehyde at room temperature. Cells were then incubated with an unconjugated goat anti-human IgG (Fc) antibody for 45 minutes (Invitrogen cat#:31125) at room temperature. Coverslips were again washed three times and incubated with Alexa Fluor 568 conjugated donkey anti-goat IgG (H+L) for 45 minutes (A11011, Invitrogen, cat#: A11011). Coverslips were washed a further four times and mounted onto 4',6-diamidino-2-phenylindole (DAPI) supplemented fluorescent mounting media on glass slides.

Coverslips were read by two independent readers using a fluorescent microscope (DM2000, Leica Microsystems) to determine the presence or absence of bound surface gnACHR antibodies. Samples were considered positive when red fluorescence was seen on the surface of the cells with the degree of fluorescence intensity graded from 1 to 4. Negative samples were considered as a score less than 1, positive samples were scored 1 to 4 based on staining intensity with 4 being the brightest. The average fluorescence scored attained by the two readers was averaged to obtain the final fluorescence score.

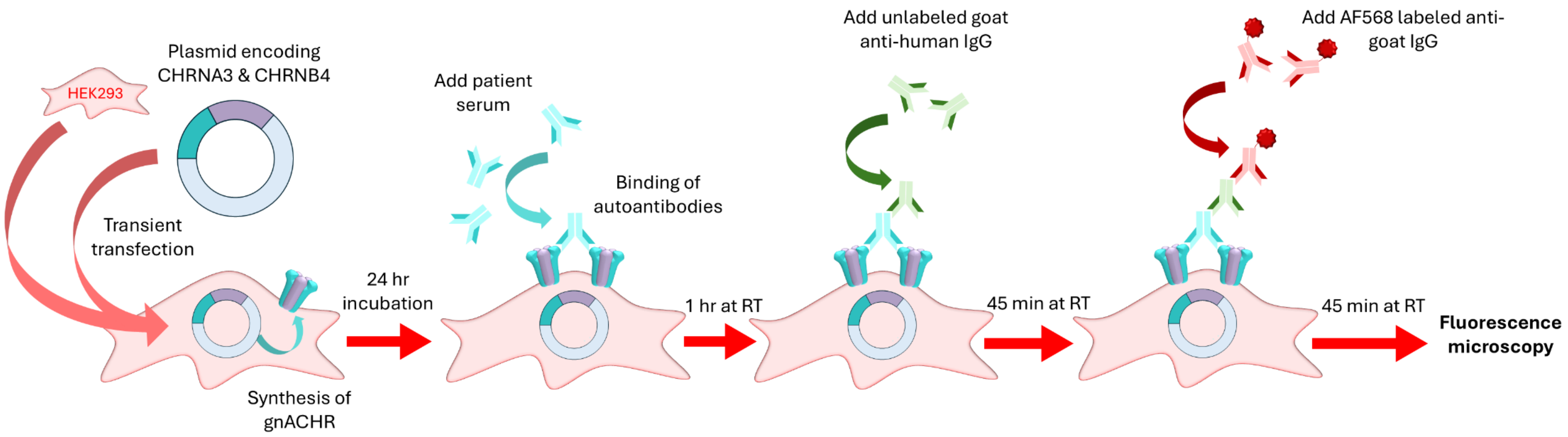

Supplementary Figure 2. Cell-based assay 1 (CBA-1) for the detection of gnACHR autoantibodies. HEK293 cells were transiently transfected with the α3 and β4 subunits of gnACHR and then incubated with patient sera. Receptor bound antibodies were detected using fluorescence labelled secondary antibodies under fluorescence microscopy.

3. Cell-based assay 2 (CBA-2)

Detection of gnACHR antibodies by CBA-2 was performed via a previously published method and is outlined in **Supplementary Figure 3** below [22].

HEK293 cells were maintained in DMEM containing 10% FBS and 1% penicillin-streptomycin at 37°C in 5% CO_2. Cells were transiently co-transfected with a mixture of plasmids for the α3 and β4 gnACHR subunits, RIC3 and NACHO subunits (pCMV6-XL4-CHRNA3 or pCMV6-XL5-CHRNA4, pCMV6-XL5-CHRNB4 or pCMV6-XL5-CHRNB2, pCMV6-XL5-TMEM35 (NACHO), and pCMV6-XL5-RIC3 (OriGene, Germany); 3.7 µg/plasmid for α3β4 or α3β2 and 2.5 µg for α4β2 per 100 mm culture dish; or control vector, using jetPRIME kit transfection reagent (Polyplus jetPRIME, France)). Cells were then treated with 1 mM nicotine (Sigma, cat#:N3876) for 24 hours before further analysis. Co-incubation with nicotine has previously been demonstrated to increase surface expression of nicotinic acetylcholine receptors and experiments were performed to determine the optimum nicotine concentration for receptor expression.

Forty-eight hours post-transfection, cells were washed in DMEM/0.46% w/v N-(2-hydroxyethyl)-piperazine-N'-(2-ethanesulfonic acid) (HEPES) buffer (DMEM-HEPES). Cells were then incubated with patient sera at 1:10 dilution for 1 hour at room temperature before being washed three times and fixed in 4% paraformaldehyde for 10 minutes. Fixed cells were incubated with rabbit anti-human IgG (Invitrogen) at 1:750 dilution for 1 hour at room temperature, followed by incubation with Alexa Fluor-568 goat anti-rabbit IgG (H + L) (Invitrogen, cat#:A-11037) at 1:750 dilution for 1 hour at room temperature with additional wash steps between incubations. Two independent observers then determined the presence or absence of bound surface gnACHR antibodies using the Olympus microscope CKX-41, and images were analyzed using Infinity Analyze-6.5 Lumenera software. AQP4-transfected HEK293 cells were used as negative controls. This assay has previously been validated with the assessment of 14 RIPA positive AAG patients and 2674 control patients [22].

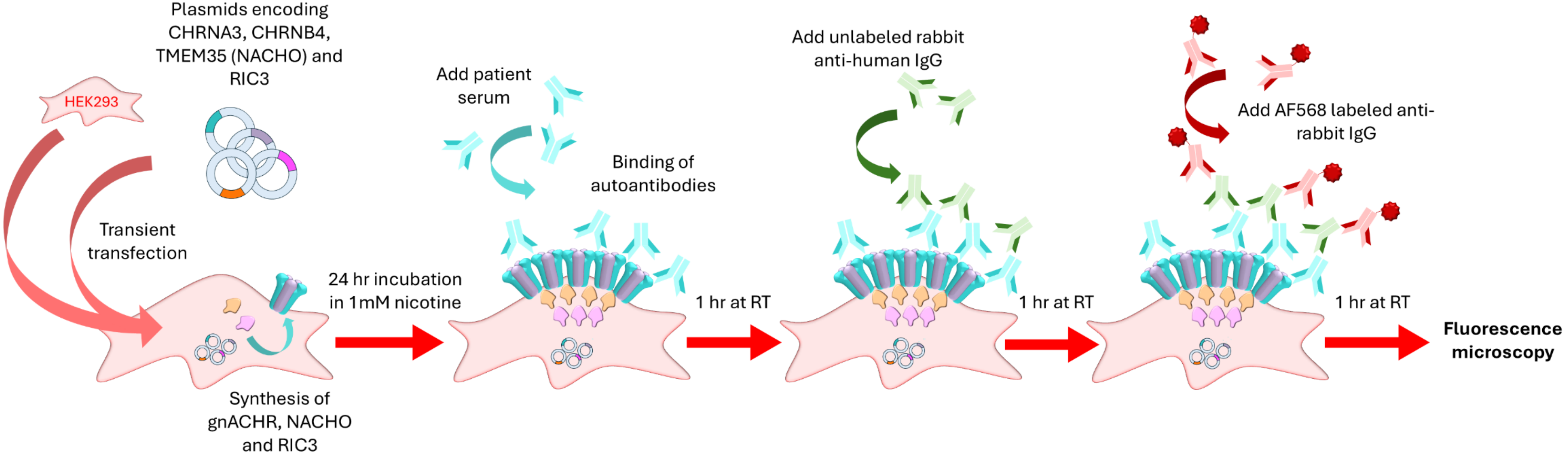

Supplementary Figure 3. Cell-based assay 2 (CBA-2) for the detection of gnACHR autoantibodies. HEK293 cells were transiently transfected with the α3 and β4 subunits of gnACHR and the chaperone proteins, RIC3 and NACHO. Cells were further incubated with 1mM nicotine to increase surface gnACHR expression. Cells were then then incubated with patient sera with receptor bound autoantibodies detected using fluorescence labelled secondary antibodies under fluorescence microscopy.

Supplementary table 1. Analytical sensitivity of the BLIA

AAG patient	Limit of detectable internalisation (pM)
Patient 1	0.05
Patient 2	0.04
Patient 3	0.07
Patient 4	0.07
Patient 5	0.05
Patient 6	0.05
Patient 7	0.03
Patient 8	0.05
Patient 9	0.05
Patient 10	0.04
Patient 11	0.05
Patient 12	0.04
Patient 13	0.05
Patient 14	0.06
Patient 15	0.05
Patient 16**	0.57
Patient 17**	1.35
Patient 18**	0.40
Patient 19**	0.29
Patient 20**	0.61

Serial dilutions of AAG patient sera were assessed to determine the limit of detection of the BLIA. Lowest autoantibody concentrations that caused gnACHR internalisation were then determined using the corresponding RIPA results. Patients 16 to 20 (**) only had RIPA results available from a prior method which utilised a different methodology and cut-off for positivity.

Supplementary table 2. Sensitivity and specificity of the BLIA and flow cytometric internalisation assay (FCIA)

Assay	Sensitivity (n = 46)	Specificity (n = 84)
Bioluminescent internalisation assay (BLIA)	100% (46/46)	100% (84/84)
Flow cytometric internalisation assay (FCIA)	100% (46/46)	98.8% (83/84)

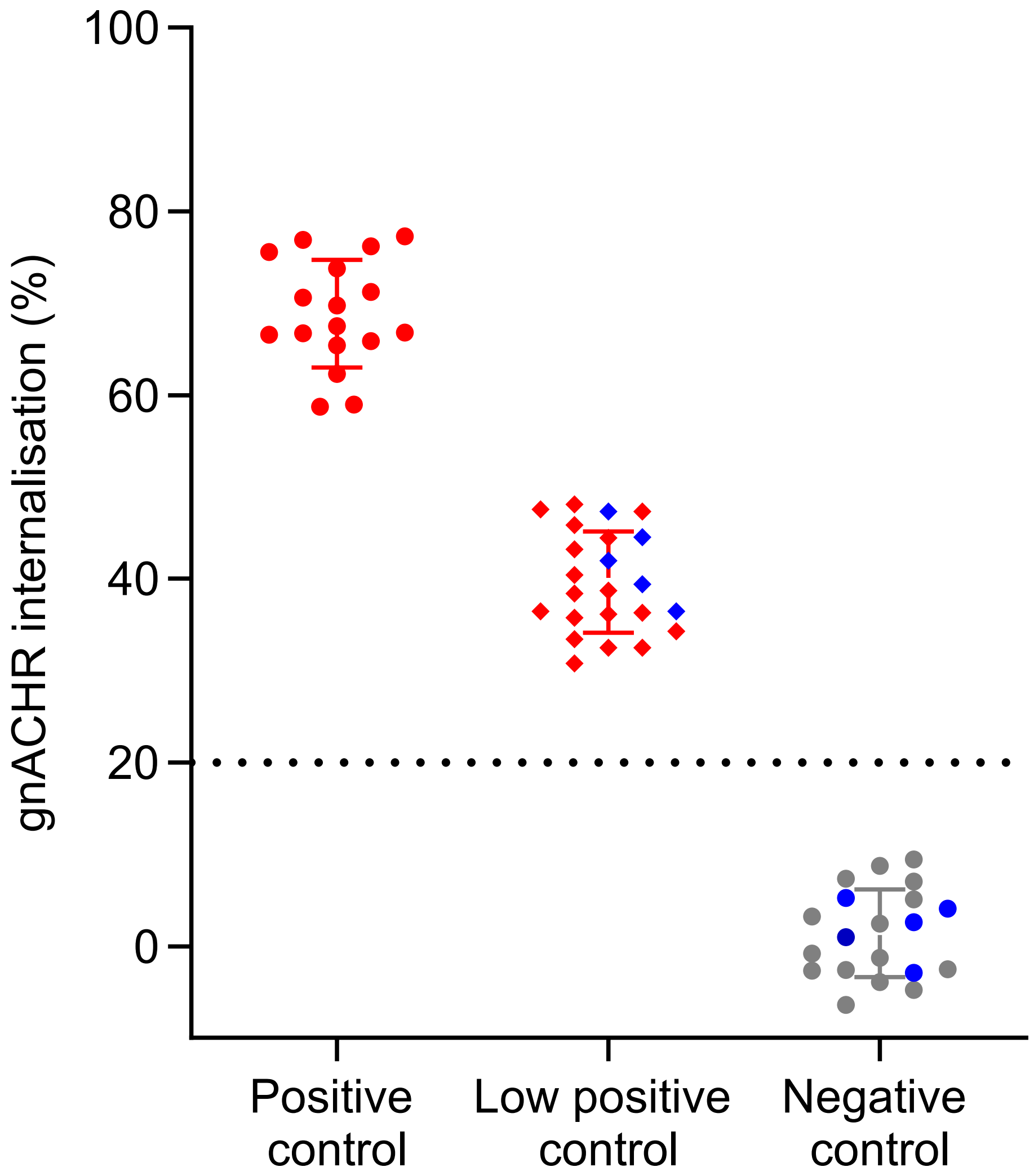

Supplementary figure 4. Inter-assay variation for the BLIA for three control samples including a positive control AAG patient (n=17), a low positive control AAG patient (n=18) and a healthy control (n=18). Standard deviations for each are shown via the error bars and were 5.8%, 5.7% and 5.2% gnACHR internalisation respectively. Values in blue denote samples with the addition of interfering substances (IVIG, lipaemic, icteric or haemolysed serum). Assay runs assessed were across different lots of IMR-32 cells, conjugated secondary antibodies and detection substrate.

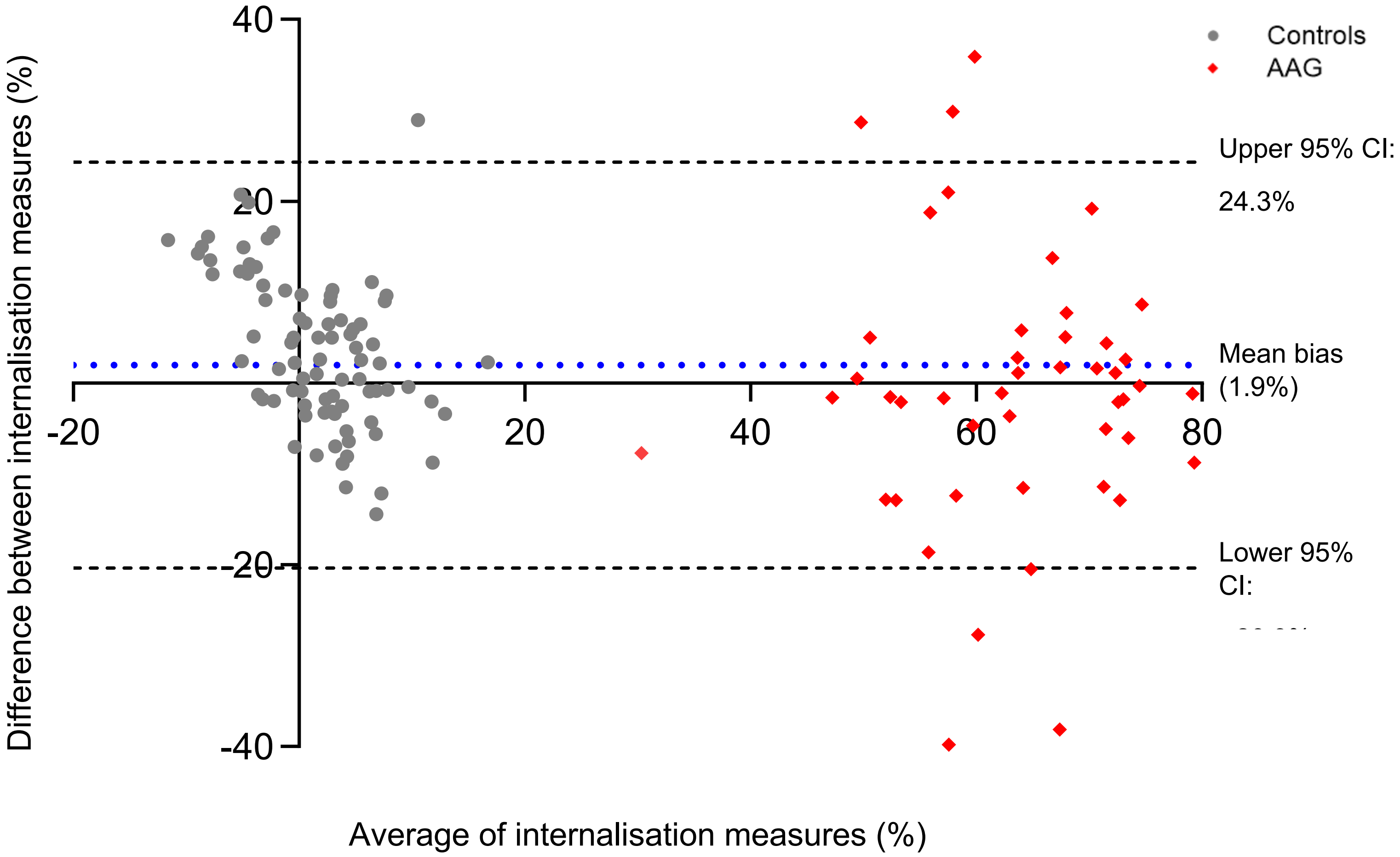

Supplementary Figure 5. Bland-Altman analysis of agreement of quantitative gnACHR internalisation (%) between the BLIA and FCIA for 130 analysed samples. The difference between gnACHR internalisation (%) obtained via the BLIA and FCIA is plotted against the average of measurements via the two assays for each sample. Positive difference values denote a higher internalisation measure via the FCIA compared to the BLIA while negative values denote a higher BLIA measurement. Calculated mean bias (dotted blue line, 1.9%) and 95% confidence intervals for mean bias (-20.3 to 24.3%, dashed black lines) are displayed.
